# A New Therapeutic Modality for Acute Myocardial Infarction: Nanoparticle-Mediated Delivery of Pitavastatin Induces Cardioprotection from Ischemia-Reperfusion Injury via Activation of PI3K/Akt Pathway and Anti-Inflammation in a Rat Model

**DOI:** 10.1371/journal.pone.0132451

**Published:** 2015-07-13

**Authors:** Kazuhiro Nagaoka, Tetsuya Matoba, Yajing Mao, Yasuhiro Nakano, Gentaro Ikeda, Shizuka Egusa, Masaki Tokutome, Ryoji Nagahama, Kaku Nakano, Kenji Sunagawa, Kensuke Egashira

**Affiliations:** 1 Department of Cardiovascular Medicine, Kyushu University Graduate School of Medical Sciences, Fukuoka, Japan; 2 Department of Cardiovascular Research, Development, and Translational Medicine, Kyushu University Graduate School of Medical Sciences, Fukuoka, Japan; Emory University, UNITED STATES

## Abstract

**Aim:**

There is an unmet need to develop an innovative cardioprotective modality for acute myocardial infarction (AMI), for which the effectiveness of interventional reperfusion therapy is hampered by myocardial ischemia-reperfusion (IR) injury. Pretreatment with statins before ischemia is shown to reduce MI size in animals. However, no benefit was found in animals and patients with AMI when administered at the time of reperfusion, suggesting insufficient drug targeting into the IR myocardium. Here we tested the hypothesis that nanoparticle-mediated targeting of pitavastatin protects the heart from IR injury.

**Methods and Results:**

In a rat IR model, poly(lactic acid/glycolic acid) (PLGA) nanoparticle incorporating FITC accumulated in the IR myocardium through enhanced vascular permeability, and in CD11b-positive leukocytes in the IR myocardium and peripheral blood after intravenous treatment. Intravenous treatment with PLGA nanoparticle containing pitavastatin (Pitavastatin-NP, 1 mg/kg) at reperfusion reduced MI size after 24 hours and ameliorated left ventricular dysfunction 4-week after reperfusion; by contrast, pitavastatin alone (as high as 10 mg/kg) showed no therapeutic effects. The therapeutic effects of Pitavastatin-NP were blunted by a PI3K inhibitor wortmannin, but not by a mitochondrial permeability transition pore inhibitor cyclosporine A. Pitavastatin-NP induced phosphorylation of Akt and GSK3β, and inhibited inflammation and cardiomyocyte apoptosis in the IR myocardium.

**Conclusions:**

Nanoparticle-mediated targeting of pitavastatin induced cardioprotection from IR injury by activation of PI3K/Akt pathway and inhibition of inflammation and cardiomyocyte death in this model. This strategy can be developed as an innovative cardioprotective modality that may advance currently unsatisfactory reperfusion therapy for AMI.

## Introduction

Coronary heart disease is the leading cause of death worldwide, and acute myocardial infarction (MI) is the most severe type of the illness [1]. Myocardial infarction (MI) size is a major determinant of clinical outcome/prognosis in patients with acute MI [2]. Reduction in MI size has been partially achieved by early reperfusion therapy with thrombolytic drugs and/or percutaneous coronary intervention in those patients. However, the restoration of blood supply to the ischemic myocardium induces ischemia-reperfusion (IR) injury, which may limit the therapeutic effects of early reperfusion [3]. Various pharmacological agents have been shown to reduce IR injury in animal models; however, none of them have been developed as cardioprotective modalities for IR injury in clinical practice [3–5]. Therefore, there is an unmet need to develop innovative cardioprotective modalities to reduce IR injury.

The 3-hydroxy-3-methylglutaryl coenzyme A reductase inhibitors (statins) are used worldwide as cholesterol-lowering drugs. In addition, statins are known to afford cardioprotection from IR injury in animals; pretreatment with statins at high doses before ischemia protects hearts from IR injury in vivo [6–8]. These unique effects of statins on limiting MI size are not related to cholesterol-lowering, but are mediated by activating pro-survival protein kinase cascades such as the PI3 kinase (PI3K)/Akt pathway and by anti-inflammatory effects. The activation of such pro-survival signaling, referred to as the “reperfusion injury salvage kinase” (RISK), pathway [9] attenuates reperfusion-induced necrosis and apoptosis, and thus reduces MI size, when administrated before IR. However, these MI-limiting effect was not replicated in animal models when administered at the time of reperfusion [6,10], which is clinically feasible time point of adjunctive therapeutic intervention upon reperfusion therapy for acute MI. In a recent placebo-controlled randomized clinical trial, pretreatment with atorvastatin (80 mg) 10–30 min before primary PCI for ST-elevation acute MI patients failed to improve cardiac function, microvascular perfusion and to decreased MI size [11]. This discrepancy in the efficacy of statins before ischemia and at reperfusion is attributed to insufficient local concentrations when administered at the time of reperfusion.

To address this challenge, we developed a nanoparticle-mediated drug delivery system using bioabsorbable poly(lactic acid/glycolic acid) (PLGA) nanoparticle. We recently reported that nanoparticle-mediated delivery of pitavastatin showed significant therapeutic effects on ischemia-induced neovascularization [12,13], pulmonary arterial hypertension [14] and restenosis [15] in animal models. Because the nanoparticle accumulates into diseased tissues/organs including IR myocardium via increased vascular permeability [16,17], we hypothesized that nanoparticle-mediated targeting of statins to IR myocardium can be a novel therapeutic modality for IR injury.

In the present study, we used a rat IR model and tested the hypothesis that (1) nanoparticles are selectively delivered to IR myocardium after intravenous injection at the time of reperfusion and (2) nanoparticle-mediated targeting of pitavastatin protects the heart from IR injury as seen by reduction in MI size and improvement of left ventricular (LV) function.

## Material and Methods

### Preparation of PLGA nanoparticle

PLGA with an average molecular weight of 20,000 and a copolymer ratio of lactide to glycolide of 75:25 (Wako Pure Chemical Industries Ltd, Osaka, Japan) was used as a matrix for the nanoparticle, whereas polyvinylalcohol (PVA-403; Kuraray, Osaka, Japan) was used as a dispersing agent. PLGA nanoparticle incorporating fluorescent marker fluorescein-isothiocyanate (FITC; Dojin Chemical, Tokyo, Japan) (FITC-NP) or pitavastatin (Kowa Pharmaceutical Co Ltd, Tokyo, Japan) (Pitavastatin-NP) was prepared by an emulsion solvent diffusion method in purified water as previously described.[12–15,18–21] The FITC-NP and Pitavastatin-NP contained 4.2% (wt/vol) FITC and 12.0% (wt/vol) pitavastatin, respectively. A sample of nanoparticle suspension in distilled water was used for particle size analysis. The diameters of FITC-NP and Pitavastatin-NP were 231 nm and 159 nm, respectively. Surface charge (zeta potential) was also analyzed by Zetasizer Nano (Sysmex, Hyogo, Japan) and was anionic [-16.7 mV (FITC-NP) and -4.1 mV (Pitavastatin-NP)].

### Experimental myocardial IR model, quantification of MI size, and protocols

**Fig 1:**
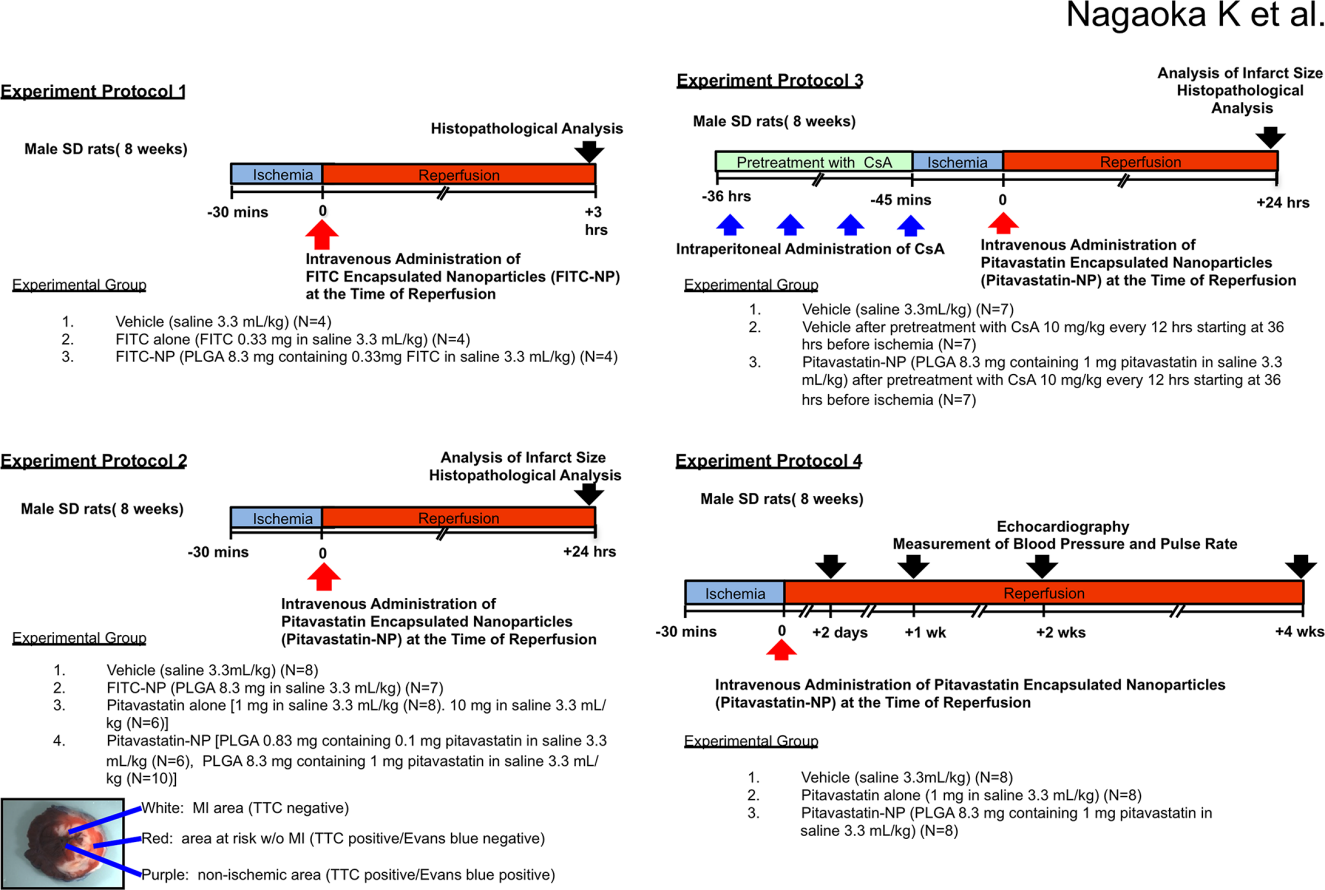
Experimental protocols. Adult male Sprague-Dawley (SD) rats, 8 weeks of age were used. Experimental protocol 1: At the time of reperfusion, animals were divided into 3 groups receiving intravenous injection of the following drugs; 1) vehicle (saline 3.3 mL/kg), 2) FITC alone (FITC 0.33 mg in saline 3.3 mL/kg), or 3) FITC-NP (PLGA 8.3 mg containing 0.33 mg FITC in saline 3.3 mL/kg). Three hours after reperfusion, animals were sacrificed. The left lower panel shows representative stereomicrographs of heart sections double-stained with Evans blue and TTC: the MI area (TTC negative, white), non-MI area within AAR (TTC positive/Evans blue negative, red), non-ischemic area (TTC positive/Evans blue positive, purple) and AAR (Evans blue negative). Experimental protocol 2: At the time of reperfusion, animals were divided into 4 groups receiving intravenous injection of the following drugs; 1) vehicle (saline 3.3 mL/kg), 2) FITC-NP (PLGA 8.3 mg/kg in saline 3.3 mL/kg), 3) pitavastatin (1.0 and 10 mg/kg in saline 3.3 mL/kg), or 4) pitavastatin-NP (PLGA containing of 0.1 and 1.0 mg/kg pitavastatin in saline 3.3 mL/kg). Twenty-four hours after reperfusion, animals were sacrificed and infarct size was measured. Experimental protocol 3: Animals were divided into 3 groups receiving administration of the following drugs; 1) vehicle (saline 3.3 mL/kg), 2) vehicle (saline 3.3 mL/kg) after pretreatment with Cyclosporine A (CsA) (10 mg/kg) every 12 hours starting 36 hours before ischemia, 3) pitavastatin-NP (PLGA containing of 1.0 mg/kg pitavastatin in saline 3.3 mL/kg) after pretreatment with CsA (10 mg/kg) every 12 hours starting 36 hours before ischemia. Twenty-four hours after reperfusion, animals were sacrificed and infarct size was measured. Experimental protocol 4: To examine the effects of Pitavastatin-NP on left ventricular function after IR, animals were divided into 3 groups that received intravenous injection of the following drugs at the time of reperfusion: 1) vehicle (saline 3.3 mL/kg), 2) pitavastatin alone (1.0 mg/kg in saline 3.3 mL/kg) or 3) Pitavastatin-NP (PLGA containing 1.0 mg/kg pitavastatin in saline 3.3 mL/kg). Echocardiography and measurement of systolic blood pressure and heart rate by using tail-cuff method were performed at baseline and 2-day, 1-week, 2-weeks and 4-weeks after reperfusion.

All experiments were reviewed and approved by the committee on ethics on animal experiments, Kyushu University Faculty of Medicine and were conducted according to the guidelines of the American Physiological Society. The myocardial IR model was based on previously described methods [10]. Briefly, adult male Sprague-Dawley (SD) rats were anesthetized with an intraperitoneal injection of sodium pentobarbital (50 mg/kg), intubated and ventilated with a respirator. Catheters were inserted into a femoral artery for measurement of systemic blood pressure using a Power Lab (AD Instruments, Castle Hill, Australia). The heart was exposed by a left thoracotomy on the heated board. Rats were subjected to myocardial ischemia by placing a 6–0 silk suture 2 mm below the tip of the left atrial appendage and making a slipknot around the left anterior descending (LAD) artery. Coronary ischemia was confirmed by epicardial cyanosis and ECG change (ST elevation). After 30 minutes or 45 minutes of ischemia, the slipknot was released to achieve reperfusion. The chest was then closed, and all animals were allowed to recover from the surgery. The 4 sets of animal experiments are depicted in a schematic form in Fig 1. For analyses, animals were sacrificed with an intraperitoneal injection of overdose of sodium pentobarbital (150 mg/kg).

Experimental protocol 1:To examine the distribution of nanoparticles; rats were subjected to myocardial ischemia for 30 minutes, followed by coronary reperfusion. At the time of reperfusion, animals were divided into 3 groups that received intravenous injection of the following drugs: 1) vehicle (saline 3.3 mL/kg), 2) FITC alone (FITC 0.33mg in saline 3.3mL/kg), 3) FITC-NP (PLGA 8.3 mg containing 0.33mg FITC in saline 3.3 mL/kg). Three hours after reperfusion, animals were sacrificed and histopathological analysis was performed.

Experimental protocol 2: To examine the effects of Pitavastatin-NP on infarct size after IR, rats were divided into 4 groups that received intravenous injection of the following drugs at the time of reperfusion: 1) vehicle (saline 3.3 mL/kg), 2) FITC-NP (PLGA 8.3 mg/kg in saline 3.3 mL/kg), 3) pitavastatin alone (1.0 and 10 mg/kg in saline 3.3 mL/kg) or 4) Pitavastatin-NP (PLGA containing 0.1 and 1.0 mg/kg pitavastatin in saline 3.3 mL/kg). In another set of experiments, wortmannin (16 μg/kg; Sigma Aldrich), the PI3K inhibitor, was intravenously administered 15 minutes before reperfusion, as previously described [22]. At the time of reperfusion, rats were treated with intravenous injection of vehicle (saline) or Pitavastatin-NP containing 1.0 mg/kg pitavastatin. Twenty-four hours after reperfusion, rats were sacrificed and infarct size was measured.

Experimental protocol 3: To examine the effects of Pitavastatin-NP on cytochrome c leakage from mitochondria into the cytosol after reperfusion, rats were divided into 3 groups receiving administration of the following drugs; 1) vehicle (saline 3.3 mL/kg), 2) vehicle (saline 3.3 mL/kg) after pretreatment with Cyclosporine A (CsA) (10 mg/kg) every 12 hours starting 36 hours before ischemia, 3) Pitavastatin-NP (PLGA containing of 1.0 mg/kg pitavastatin in saline 3.3 mL/kg) after pretreatment with CsA (10 mg/kg) every 12 hours starting 36 hours before ischemia. Twenty-four hours after reperfusion, rats were sacrificed and infarct size was measured.

Experimental protocol 4: To examine the effects of Pitavastatin-NP on left ventricular function after IR, rats were divided into 3 groups that received intravenous injection of the following drugs at the time of reperfusion: 1) vehicle (saline 3.3 mL/kg), 2) pitavastatin alone (1.0 mg/kg in saline 3.3 mL/kg), or 3) Pitavastatin-NP (PLGA containing 1.0 mg/kg pitavastatin in saline 3.3 mL/kg). Echocardiography and measurement of systolic blood pressure and heart rate by using tail-cuff method were performed at baseline and 2 days, 1 week, 2 weeks and 4 weeks after reperfusion.

### Quantification of MI size

Twenty-four hours after reperfusion, rats were anesthetized with an intraperitoneal injection of sodium pentobarbital and were intubated. The LAD was re-occluded, and 4% Evans blue dye (Sigma Aldrich) was injected via the inferior vena cava to identify area at risk (AAR). The heart was then excised and perfused with saline and cut into sequential 2-mm-thick cross sections. The sections were incubated with 1% 2,3,5-triphenyltetrazolium chloride (TTC, Sigma Aldrich) for 10 minutes at 37°C and were photographed with a stereomicroscope (Nikon, HC-2500). The MI area (TTC negative, white), non-MI area within AAR (TTC positive/Evans blue negative, red), non-ischemic area (TTC positive/Evans blue positive, purple), and AAR (Evans blue negative) were analyzed using ImageJ software (version 1.44) (Fig 1).

### Distribution of FITC-NP after intravenous injection

Distribution of FITC in the heart was examined. After myocardial IR, animals were sacrificed, and organs were then harvested and fixed in 10% phosphate-buffered formalin (pH 7.4). Distributions of FITC-NP were analyzed in 5-μm OCT-embedded sections or 5-μm paraffin sections.

### Measurements of pitavastatin concentrations in plasma and heart tissue

Pitavastatin concentrations in plasma and the heart were measured at predetermined time points by liquid chromatography coupled to tandem mass spectrometry (LC/MS/MS). Briefly, the high-performance liquid chromatography (HPLC) analysis was performed using Agilent 1100 series system (Agilent Technologies, Inc, Santa Clara, CA, USA). The column temperature was maintained at 40°C. The flow rate was 0.3 mL/min. Pre-prepared plasma or tissue homogenate sample solutions were injected from the autosampler into the HPLC system. The turbo ion spray interface was operated in the positive ion mode at 4800 V and 550°C. The analytical data were processed using Analyst software (version 1.4, Applied Biosystems, Foster City, CA, USA)

### Measurement of vascular permeability

Vascular permeability was measured by dye extraction method as previously described [23,24]. In brief, Evans blue dye diluted in saline at 30 mg/mL was injected via femoral vein 2.5 hours after reperfusion. Thirty minutes after dye injection, the heart was harvested and perfused with 10 mL saline through a catheter passed through the ascending aorta. To measure the extravasated Evans blue, IR myocardium and non-ischemic myocardium were isolated and weighed. They were kept in formamide, 4 mL/g•tissue at room temperature for 24 hours. Permeability was quantitated by measuring the amount of Evans blue dye extracted in formamide by spectrophotometry at a wavelength of 595 μm.

### Western blot analysis

Homogenate of IR myocardium was analyzed with immunoblotting. At predetermined time points, ischemic myocardium was isolated and analyzed as previously reported [25]. Briefly, frozen samples were homogenized in lysis buffer and proteins (3, 5, or 20 μg) were separated on 7.5 or 15% SDS-polyacrylamide gels and then blotted to PVDF membranes. The following antibodies were used as primary antibodies: p-Akt (Ser473, 1:2000, #4058, Cell Signaling), Akt (1:2000, #9272, Cell Signaling), p-GSK3β (Ser9, 1:1000, #9336, Cell Signaling), GSK3β (1:1000, #9315, Cell Signaling), cytochrome C (cytosol fraction; 1:1000, mitochondrial fraction; 1:2000, A8 sc13156, Santa Cruz Biotechnology), VDAC (1:1000, #4661S, Cell Signaling), GAPDH (1:2500, sc-20357, Santa Cruz Biotechnology), and α-tubulin (1:1000, sc-8035, Santa Cruz Biotechnology). Densitometoric analyses were performed using Image J software (version 1.44).

### Isolation of rat heart mitochondria

Rat heart mitochondria were isolated according to the manufacturer’s protocol (Abcam) [26]. Thirty minutes after IR, rats were anesthetized, and the heart was quickly excised. The IR myocardium was isolated, minced on ice, resuspended in isolation buffer and homogenized with a glass Dounce homogenizer and Teflon pestle. Homogenates were centrifuged at 1,000 × *g* for 5 minutes at 4°C. The supernatant was re-centrifuged at 12,000 × *g* for 15 minutes to pellet the mitochondria, twice. The supernatant was re-centrifuged and resultant supernatant was used as cytosolic fraction.

### Mitochondria swelling assay

Mitochondria (0.5 mg/ml) isolated from cardiac samples taken 10min after the onset of reperfusion were suspended in a buffer containing (250 mmol/L sucrose 10 mmol/L MOPS, 5 mmol/L EGTA, 2 mmol/L MgCl2, 5 mmol/L pyruvate, and 5 mmol/L malate) and incubated with 150 mol/L calcium chloride (CaCl2) in a final volume of 200 mL in a 96-well plate for 20 min. Mitochondrial swelling was assessed spectrophotometrically as a decrease in absorbance at 520 nm (A_520_) [27].

### Flow cytometry

Peripheral blood was drawn via cardiac puncture, and red blood cells were lysed with VersaLyse Lysing solution (Becton Dickinson Bioscience, San Jose, California) for 10 minutes at room temperature. Hearts were removed and digested with a cocktail of 450 U/mL collagenase type I, 125 U/mL collagenase type XI, 60 U/mL DNase I and 60 U/mL hyaluronidase (all enzyme were obtained from Sigma-Aldrich) in PBS containing 20 mM Hepes at 37°C for 1 hr. The cell suspension was centrifuged at 300 x g for 5 minutes at 4°C. After blocking the Fc receptor, cell suspensions were incubated for 1h at 4°C. The leukocytes were also incubated with isotype controls. Leukocytes from peripheral blood and the heart were analyzed with Gallios (Beckman Coulter, Inc.).

### Histology

Three or 24 hours after reperfusion, hearts were harvested and fixed overnight in 10% buffered-formalin. After fixation, the tissue was embedded in paraffin or frozen with OCT compound. Serial cross sections (5-μm thick) were used for analysis. The sections were subjected to immunohistostaining using anti-ED-1 antibodies (1:500, Dai-nippon Pharma), anti-MCP-1 antibodies (1:200, Santa Cruz Biotechnology), anti-FITC antibodies (1:1000. American Research Products), anti-Troponin T antibodies (1:500, Thermo Scientific), anti-p-Akt antibodies (1:50, Cell Signaling), and anti-p65 subunit of NF-B (1:100, Roche Diagnostics). The degrees of macrophage infiltration and MCP-1 expression in the AAR 24 hours after reperfusion were evaluated. Digital images of 5–10 fields in the AAR per heart were stored, and the number of infiltrated macrophages and NF-B positive cells, and MCP-1 staining area were counted using a 40 × objective, as previously described [28]. The rat heart was harvested after 24 hours of reperfusion and fixed. The heart was embedded in paraffin. Apoptotic nuclei were detected by terminal deoxynucleotidyl-transferase mediated dUTP nick-end labeling (TUNEL) staining using an in situ apoptosis detection kit (Takara Bio, Inc., Shiga, Japan) as previously described [29]. The percentage of TUNEL-positive myocytes was counted in the border area and determined on 1,000 total nuclei using the 40× objective. MI scar size, fibrotic area, and myocyte cross-sectional area were measured as previously described [30].

### Echocardiography

Rats were anesthetized with inhaled isoflurane (1–2%; Abbott) and placed in supine position on a warming pad. Using a Vevo 2100 high-frequency, high-resolution digital ultrasound system (Primetech Inc) and 18 MHz transducer (MS 200), 2-dimentional echocardiographic measurements of left ventricular function were performed at baseline and 2 days, 1 week, 2 weeks, and 4 weeks after reperfusion. Short axis values of left ventricular (LV) end-diastolic diameter (LVEDD) and LV end-systolic diameter (LVESD) were obtained by M-mode tracings and LV ejection fraction (EF) and LV fractional shortening (FS) were calculated. An average of three consecutive cardiac cycles was used for each measurement and was made three times in an investigator-blinded manner.

### Statistical analysis

Data are expressed as the mean±SEM. The statistical analysis of differences between two groups was assessed with the unpaired t-test, and the differences among more than three groups were assessed by ANOVA and multiple comparison tests with Prism Software version 4.0 (GraphPad Software, San Diego California USA). P values <0.05 were considered to be statistically significant.

## Results

### Distribution of PLGA-nanoparticles *in vivo*

**Fig 2:**
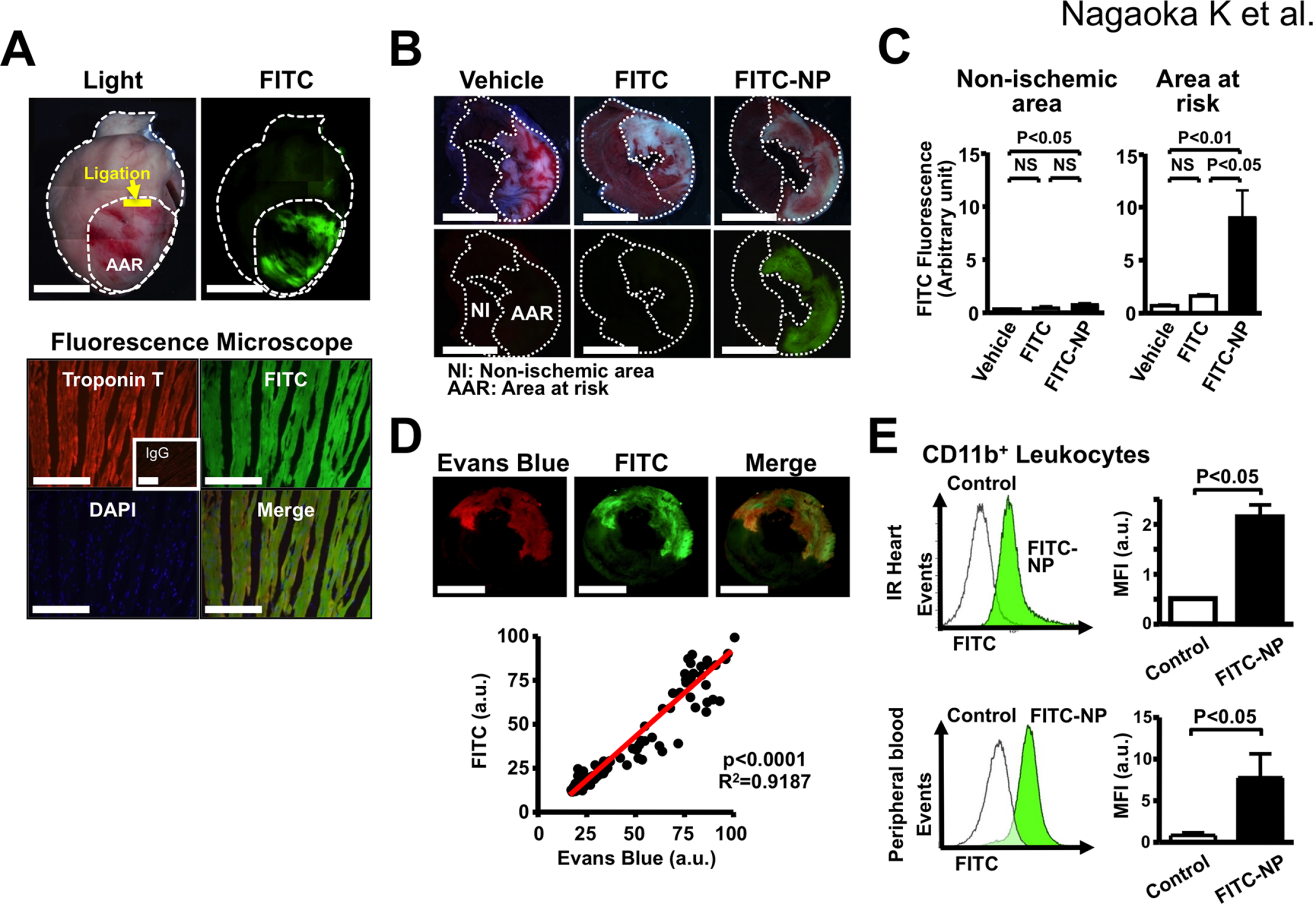
Distribution of nanoparticles in rats 3-hour after intravenous injection of FITC-NP. **(A),** Representative light (left) and fluorescence (right) stereomicrographs of whole hearts 3 hours after intravenous injection of FITC-NP. Scale bar: 5 mm (upper). Fluorescence microscopic images of the cross sections of the IR hearts treated with FITC-NP. Cardiomyocytes are identified by anti-troponin T antibody (red) and nuclei by DAPI (blue). Merged image shows colocalization of troponin T and FITC-NP. Scale bar: 100 nm (lower). **(B)**, Representative light (upper) and fluorescence (lower) stereomicrographs of cross-sections of the IR hearts 3-hour after intravenous injection of vehicle, FITC alone, or FITC-NP. In the light images, hearts were double-stained with Evans blue and TTC to determine the area at risk. Scale bar: 5 mm. **(C)**, Quantification of FITC fluorescence intensity in AAR and non-ischemic area 3-hour after intravenous injection of vehicle, FITC alone, or FITC-NP. N = 4 each. Data are compared using one-way ANOVA followed by Bonferroni’s multiple comparison tests. **(D)**, Fluorescence stereomicrographs of the IR hearts from rats co-treated with Evans blue dye and FITC-NP. Evans blue (red) and FITC (green) fluorescence signals were co-localized in IR myocardium. Scale bar: 5 mm. Close correlation between the intensity of FITC and Evans blue. Thirty ROIs were placed on the fluorescence images of heart sections per animal (n = 4) at random. Values of mean fluorescence intensity of both FITC and Evans blue were determined in the same ROI (n = 120). Pearson’s correlation was used to investigate relationships between the fluorescence intensity of FITC and Evans blue. **(E)**, Flow cytometric histograms of CD11b-positive leukocytes in the IR hearts and the blood 24-hour after intravenous injection of FITC-NP. Cells were labeled with anti-CD11b antibodies. White indicates control fluorescence in cells derived from uninjected animals. Green indicates fluorescence in cells derived from FITC-NP injected mice. Right graphs show mean fluorescence intensity (MFI) in CD11b-positive leukocytes in ischemic myocardium (upper) and blood (lower). Data are mean±SEM (n = 5 per group). Data are compared using unpaired t tests.

We examined the distribution of FITC in the heart. Strong FITC signals were detected in ischemic area (AAR) from the IR hearts of rats injected with FITC-NP at reperfusion (Fig 2A). Immunofluorescence analysis confirmed the presence of FITC signals in troponin T-positive cardiomyocytes (Fig 2A). We then prepared cross-sections of the IR hearts and examined tissue distribution of FITC in the area at risk (AAR) and the non-ischemic area after the treatment with FITC alone or FITC-NP. Significant FITC signals were detected in the AAR 3 hours after treatment with FITC-NP, whereas no detectable fluorescence signals were found after treatment with the same amount of FITC alone or vehicle, or non-ischemic area after the treatment with vehicle, FITC alone, or FITC-NP (Fig 2B and 2C), suggesting an enhancement of FITC delivery by PLGA nanoparticle into IR myocardium. No significant FITC signals were noted in intact hearts from animals injected with FITC or FITC-NP without IR procedure (data not shown). In IR hearts from animals injected intravenously with FITC-NP and with Evans blue, we examined the localization of FITC and Evans blue and found that the distribution of Evans blue was closely correlated with the distribution of FITC (Fig 2D), suggesting a role of enhanced vascular permeability in the mechanism of PLGA nanoparticle delivery to the IR cardiomyocytes [23]. Flow cytometry of the IR heart and blood 24-hour after IR and FITC-NP treatment revealed that CD11b-positive leukocytes in the IR heart and blood had significant FITC signals (Fig 2E), suggesting that PLGA nanoparticle were delivered to CD11b-positive leukocytes in the circulation and in the IR myocardium.

Plasma and tissue concentrations of pitavastatin were measured in IR animals (Table 1). In the Pitavastatin-NP group, the myocardial concentrations of pitavastatin were 2- to 3-fold higher in IR myocardium than in non-ischemic myocardium at 30 min, 3 hours, and 24 hours of reperfusion. There were no differences in myocardial concentrations of pitavastatin in IR myocardium between pitavastatin and pitavastatin-NP groups at 30 min, 3 hours, and 24 hours of reperfusion.

**Table 1:** Plasma and tissue concentrations of pitavastatin after intravenous administration of pitavastatin-NP or pitavastatin at time of reperfusion.

**Pitavastatin-NP containing 1.0 mg/kg pitavastatin**			
Groups	Time after intravenous administration		
	30 minutes	3 hours	24 hours
Ischemic myocardial area at risk	236 ± 32 #	48 ± 8 #	6 ± 1#
Non-ischemic myocardium	134 ± 17	17 ± 4	3 ± 1
Lung	194 ± 31	28 ± 3	2 ± 1
Plasma	302 ± 28*	86 ± 17	4 ± 1
**1.0 mg/kg pitavastatin**			
Groups	Time after intravenous administration		
	30 minutes	3 hours	24 hours
Ischemic myocardial area at risk	256 ± 79	22 ± 3	31 ± 23
Non-ischemic myocardium	260 ± 79	11 ± 2	35 ± 25
Lung	184 ± 29	55 ± 32	9 ± 8
Plasma	214 ± 11	60 ± 8	6 ± 2

Data are expressed as the mean±SEM (ng/g•tissue for myocardial or lung tissue, and ng/mL for plasma, n = 6 each).

*P<0.05 versus pitavastatin group.

*#P*<0.05. versus non-ischemic myocardium by the unpaired t-test.

Values below measurable limits are replaced with the one-half value of the limits. The statistical analysis of differences between two groups, in which one of values was replaced by the complementary value, was assessed with the unpaired *t*-test after transforming values into a natural logarithm.

Plasma concentrations of pitavastatin were significantly higher in the Pitavastatin-NP group than in the pitavastatin group 30 min after reperfusion (Table 1). There were no differences in pitavastatin concentrations in the lung between the Pitavastatin-NP and pitavastatin groups.

### Effects of Pitavastatin-NP on MI size

**Fig 3:**
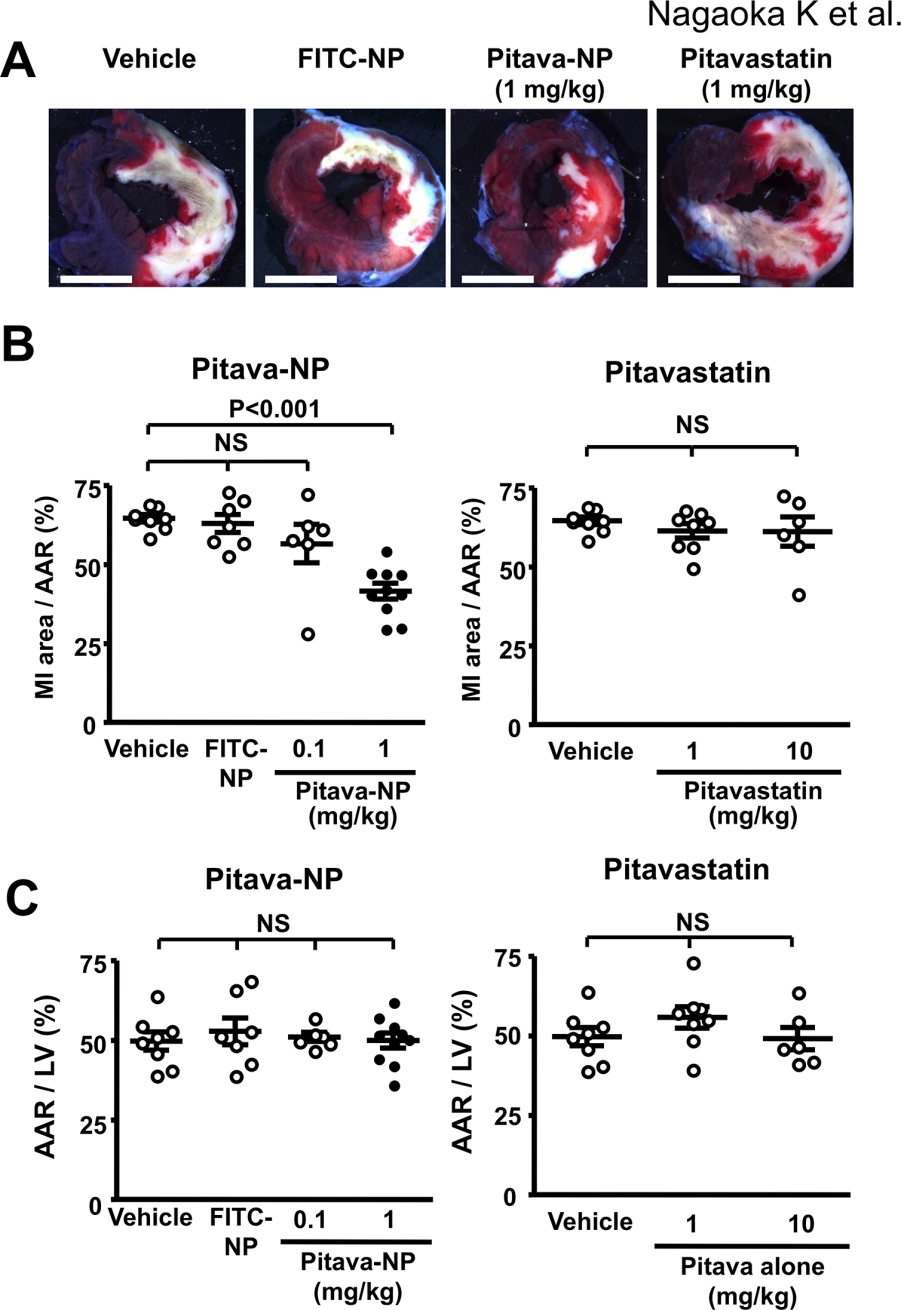
Effects of Pitavastatin-NP on MI size. **(A)**, Representative stereomicrographs of heart sections double-stained with Evans blue and TTC 24 hours after reperfusion. Scale bar: 5 mm. **(B),** Effects of Pitavastatin-NP and pitavastatin alone on MI size at the time of reperfusion. N = 6–10 per group. Data are compared using one-way ANOVA followed by Bonferroni’s multiple comparison tests. **(C)**, Quantification of Area at risk in the group treated with pitavastatin-NP or pitavastatin alone. Data are mean are (n = 6–10 per group) Data are compared using one-way ANOVA followed by Bonferroni’s multiple comparison tests.

Intravenous treatment with Pitavastatin-NP containing pitavastatin 1 mg/kg at the time of reperfusion significantly reduced MI size 24 hours after reperfusion (Fig 3A and 3B). FITC-NP was used as a control and showed no effects on MI size. As previously reported by other groups using rosuvastatin [7] or fluvastatin [10], intravenous treatment with pitavastatin at 1 and 10 mg/kg at the time of reperfusion did not reduce MI size (Fig 3A and 3B). Treatment with Pitavastatin-NP or pitavastatin alone did not affect AAR in the hearts (Fig 3C), plasma biochemical data except CPK (Table 2), or hemodynamic parameters (Table 3). Decreased plasma CPK levels in the Pitavastatin-NP group are consistent with therapeutic effects of Pitavastatin-NP on MI size (Table 2).

**Table 2:** Leukocyte counts and Plasma biomarker profile 24 hours after IR.

	Vehicle	FITC-NP	Pitava alone	Pitavastatin-NP
WBC (x10^2^ /μL)	76 ± 7	84 ± 19	87 ± 11	85 ± 5
CPK (IU/L)	377 ± 65	259 ± 31	339 ± 44	192 ± 26 *
Myoglobin (ng/mL)	< 10	< 10	< 10	< 10
T-Bil (mg/dl)	0.03 ± 0.01	0.03 ± 0.01	0.03 ± 0.01	0.03 ± 0.01
AST (IU/L)	566 ± 138	317 ± 61	422 ± 119	319 ± 33
ALT (IU/L)	70 ± 13	51 ± 7	74 ± 27	51 ± 5
BUN (mg/dl)	19.3 ± 1.2	17.2 ± 0.7	18.6 ± 1.0	19.0 ± 1.8
Creatinine (mg/dl)	0.26 ± 0.01	0.26 ± 0.02	0.26 ± 0.02	0.23 ± 0.02
TG (mg/dl)	69.8 ± 16.6	50.5 ± 6.5	74.2 ± 26.8	50.7 ± 5.5
Total cholesterol (mg/dl)	66 ± 5	81 ± 8	60 ± 4	67 ± 3
LDL cholesterol (mg/dl)	14 ± 1	18 ± 2	11 ± 1	15 ± 2

Data are expressed as the mean ± SEM (N = 6 each).

*P<0.05 versus vehicle group. IR: ischemia-reperfusion.

**Table 3:** Effects of Pitavastatin-NP on hemodynamic parameters.

**Mean BP(mmHg)**							
Groups	Baseline	Ischemia			Reperfusion		
		0 min	10 min	20 min	0 min	5 min	10 min
Vehicle	108±4	100±6	113±4	110±5	105±3	100±7	102±4
FITC-NP	108±6	106±3	109±6	113±6	108±6	104±5	101±6
Pitava alone	107±6	105±4	109±7	109±6	98±7	101±6	102±7
Pitavastatin-NP	102±2	100±6	108±6	107±8	104±7	103±7	105±5
**HR (bpm)**							
Groups	Baseline	Ischemia			Reperfusion		
		0 min	10 min	20 min	0 min	5 min	10 min
Vehicle	429±5	429±9	424±11	419±12	400±7	408±10	399±4
FITC-NP	423±23	440±19	420±25	440±15	421±12	431±8	407±20
Pitava alone	438±16	435±3	430±9	433±10	401±13	421±11	415±10
Pitavastatin-NP	407±18	416±12	427±11	421±13	406±14	404±13	398±14

Data are expressed as the mean ± SEM (N = 6 each).

### Effects of Pitavastatin-NP on the RISK pathway

**Fig 4:**
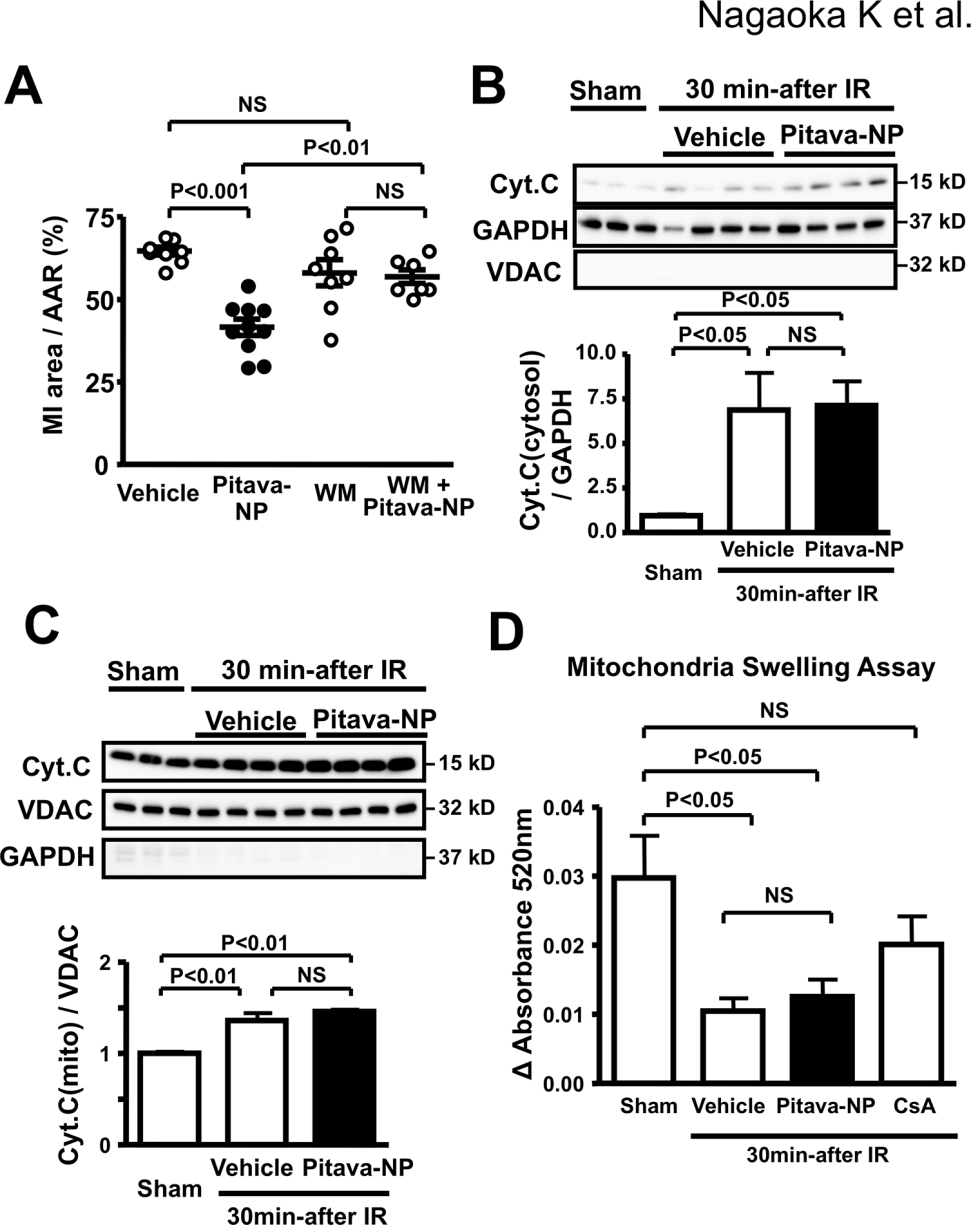
Effects of Pitavastatin-NP on mitochondrial permeability transition pore openning. **(A)**, Effects of WM on therapeutic effects of Pitavastatin-NP on MI size. Data are expressed as the mean±SEM (N = 6–10 per group). Data are compared using one-way ANOVA followed by Bonferroni’s multiple comparison tests. **(B)**, Effects of Pitavastatin-NP at the time of reperfusion on cytosolic cytochrome C in IR myocardium 30 minutes after reperfusion. N = 6 per group. Data are compared using one-way ANOVA followed by Bonferroni’s multiple comparison tests. **(C),** Effects of Pitavastatin-NP at the time of reperfusion on mitochondrial cytochrome C in IR myocardium 30 minutes after reperfusion. Data are mean±SEM (n = 6 per group). Data are compared using one-way ANOVA followed by Bonferroni’s multiple comparison tests. **(D)**, Effects of Pitavastatin-NP at the time of reperfusion on mitochondrial swelling in IR myocardium 10 minutes after reperfusion. N = 5 per group. Data are compared using one-way ANOVA followed by Bonferroni’s multiple comparison tests.

Pretreatment with wortmannin abolished the therapeutic effects of Pitavastatin-NP on MI size, whereas wortmannin had no effect on MI size in vehicle-treated animals (Fig 4A). Importantly, Pitavastatin-NP did not reduce IR-induced leakage of cytochrome C from the mitochondrial fraction into the cytosolic fraction 30 min after IR that depends on mitochondrial permeability transition pore (mPTP) opening (Fig 4B), without changes in cytochrome C in the mitochondrial fraction (Fig 4C). We performed mitochondria swelling assays to examine the effects of pitavastatin-NP on mPTP opening and found that pitavastatin-NP did not affect mitochondria swelling, while the pretreatment with cyclosporine A reduced the mitochondria swelling (Fig 4D).

**Fig 5:**
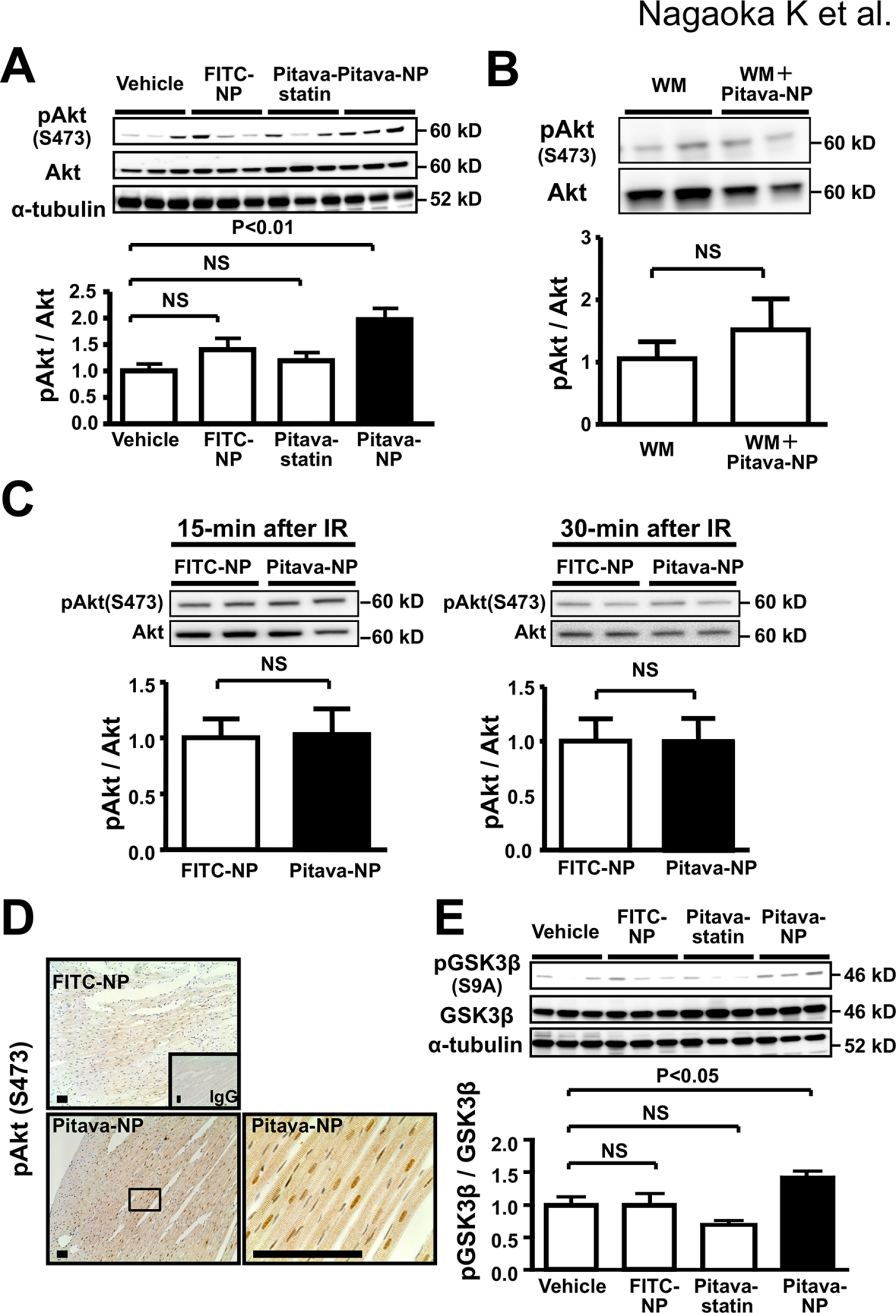
Effects of Pitavastatin-NP on RISK pathway. **(A)**, Western blot analysis of phosphorylated Akt (Ser 473) in IR myocardium 3 hours after reperfusion. N = 6 per group. Data are compared using one-way ANOVA followed by Dunnett’s multiple comparison tests. **(B)**, Western blot analysis of phosphorylated Akt in IR myocardium from animals treated with WM or with WM plus pitavastatin-NP, 3 hours after reperfusion. Data are mean±SEM (n = 6 per group) **(C)** Western blot analysis of phosphorylated Akt in IR myocardium from animals treated with FITC-NP or with pitavastatin-NP, 15 and 30 minutes after reperfusion. **(D)**, Representative photomicrographs of IR areas of hearts treated with FITC-NP (left) and Pitavastatin-NP (middle and right) stained immunohistochemically with antibody against phospho-Akt, and an expanded view of the boxed area of the middle panel (right). Scale bar: 100 μm. **(E)**, Western blot analysis of phosphorylated GSK3β (S9A) in IR myocardium 3 hours after reperfusion. N = 6 per group. Data are compared using one-way ANOVA followed by Dunnett’s multiple comparison tests.

Pitavastatin-NP (1 mg/kg) induced phosphorylation of Akt (Ser 473) 3 hours after IR in a PI3K-dependent manner (Fig 5A and 5B), but not 15 minutes and 30 minutes after IR (Fig 5C) when mPTP opening plays a role in cytochrome C leakage and myocardial necrosis [3]. Immunohistochemistry revealed that the phosphorylated Akt localized mainly in cardiomyocytes within the AAR (Fig 5D). Treatment with Pitavastatin-NP also induced GSK3β phosphorylation (S9A) 3 hours after IR (Fig 5E), suggesting that Pitavastatin-NP exert cardioprotection through the RISK pathway, but not through the inhibition of mPTP opening. Pitavastatin alone (1 mg/kg) failed to exert phosphorylation of Akt or GSK3β at 3 hours after IR (Fig 5A and 5E).

### Effects of an mPTP inhibitor cyclosporine A on cardioprotection by Pitavastatin-NP

**Fig 6:**
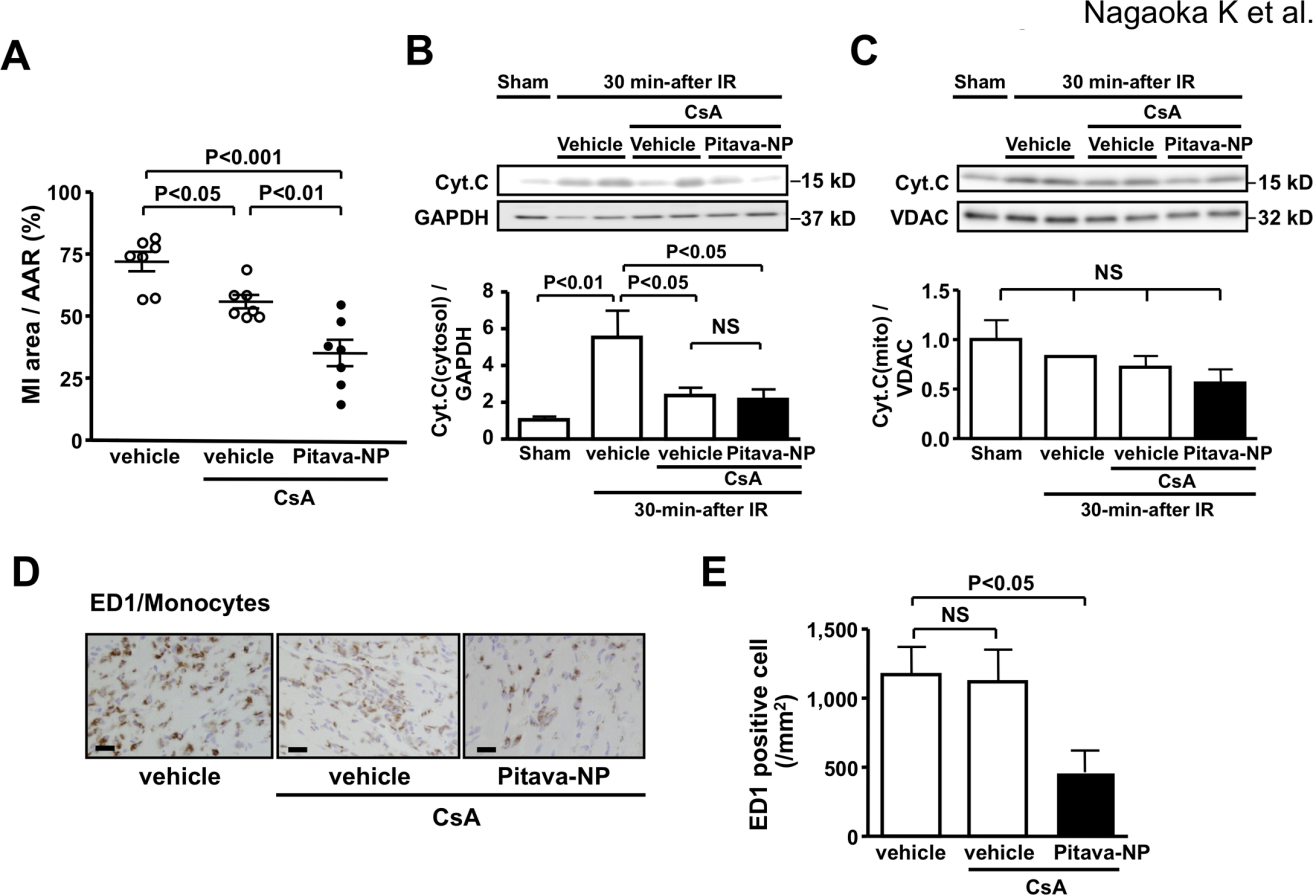
Effects of Pitavastatin-NP on cell death after IR. **(A)**, Effects of Pitavastatin-NP at the time of reperfusion after pretreatment with Cyclosporine A (CsA) (10 mg/kg) every 12 hours starting 36 hours before ischemia on MI size. N = 7 per group. Data are compared using one-way ANOVA followed by Bonferroni’s multiple comparison tests. **(B)**, Effects of Pitavastatin-NP at the time of reperfusion after pretreatment with CsA (10 mg/kg) every 12 hours starting 36 hours before ischemia on cytosolic cytochrome C in IR myocardium 30 minutes after reperfusion. N = 4 per group. Data are compared using one-way ANOVA followed by Bonferroni’s multiple comparison tests. **(C)**, Effects of Pitavastatin-NP at the time of reperfusion after pretreatment with Cyclosporine A (CsA) (10 mg/kg) every 12 hours starting 36 hours before ischemia on mitochondrial cytochrome C in IR myocardium 30 minutes after reperfusion. Data are mean±SEM (n = 4 per group). Data are compared using one-way ANOVA followed by Bonferroni’s multiple comparison tests. **(D)**, Representative photomicrographs of cross-sections from IR myocardium stained with ED-1 in AAR. Scale bar: 20 μm. **(E),** Effects of Pitavastatin-NP at the time of reperfusion after pretreatment with CsA (10 mg/kg) every 12 hours starting 36 hours before ischemia on ED-1-positive leukocyte (monocytes) infiltration in IR myocardium 24-hour after reperfusion. N = 7 per group. Data are compared using one-way ANOVA followed by Dunnett’s multiple comparison tests.

To examine the effects of Pitavastatin-NP on mPTP opening and inflammation after IR, we extended myocardial ischemia time from 30-min to 45-min, which resulted in more severe inflammation (Protocol 3). As reported by others [31,32], pretreatment with cyclosporine A, an mPTP inhibitor, significantly reduced MI size associated with reduced leakage of cytochrome C from the mitochondria into the cytosol (Fig 6A and 6B), without changes in cytochrome C in the mitochondrial fraction (Fig 6C). In animals pretreated with cyclosporine A, additional treatment with Pitavastatin-NP at reperfusion reduced MI size (Fig 6A) without further reduction of the leakage of cytochrome C (Fig 6B). Pretreatment with cyclosporine A did not affect monocyte infiltration into IR myocardium. Importantly, additional treatment with Pitavastatin-NP reduced monocyte infiltration into IR myocardium, confirming an anti-inflammatory effect of Pitavastatin-NP independent of mPTP opening. (Fig 6D and 6E).

### Effects of Pitavastatin-NP on inflammation and cardiomyocyte apoptosis

**Fig 7:**
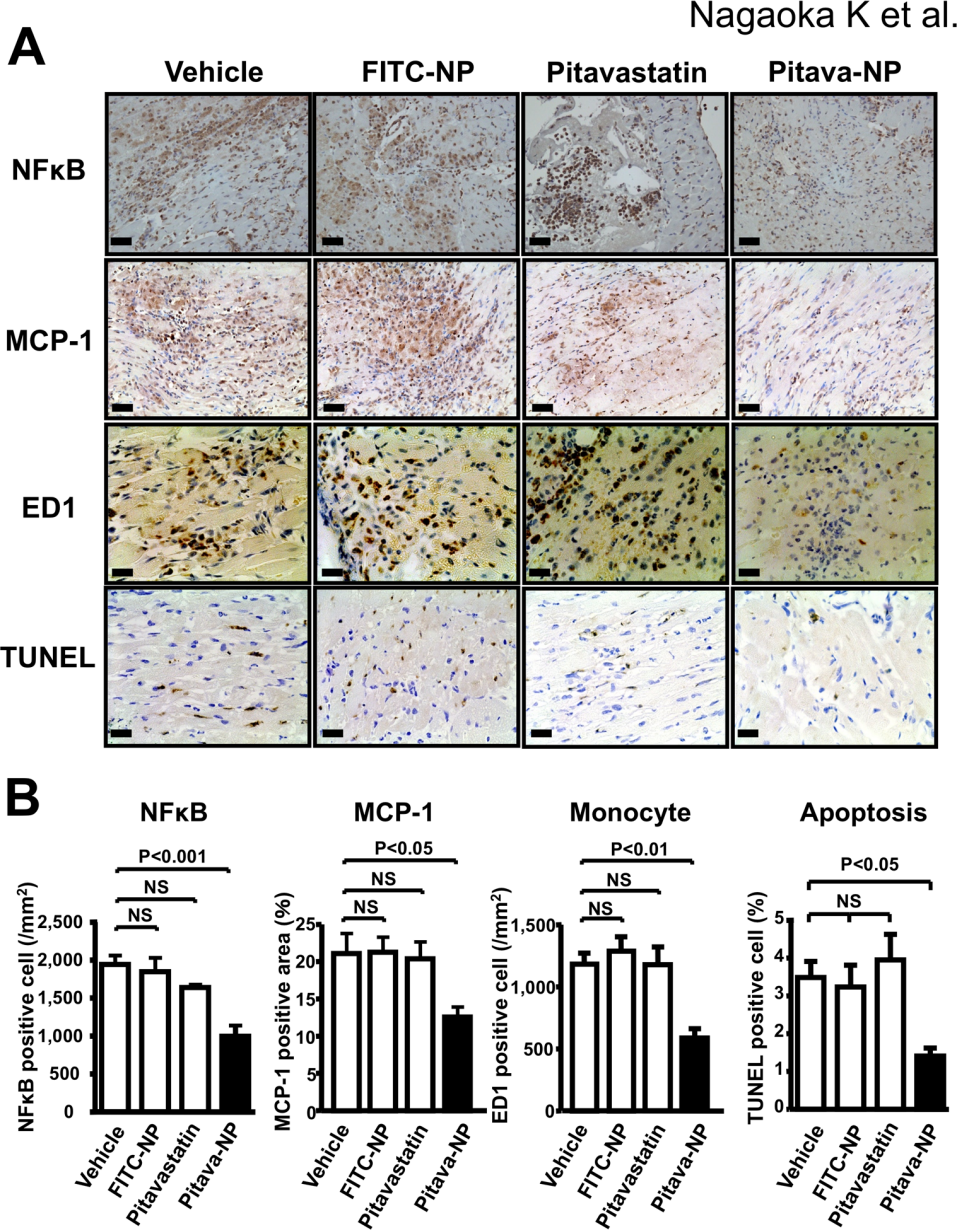
Effects of Pitavastatin-NP on inflammation and apoptosis in IR myocardium 24 hours after reperfusion. **(A)**, Representative photomicrographs of cross-sections from IR myocardium stained with NF-B (p65 subunit), MCP-1, ED-1 and TUNEL. Scale bar: 20 μm. **(B)**, Quantification of the number of NF-B (p65 subunit) positive cells, the MCP-1-positive area, ED-1-positive leukocytes (monocytes) in IR myocardium and the number of TUNEL-positive cells in infarct border myocardium 24 hours after reperfusion. Data are mean±SEM (n = 5–8 per group). Data are compared using one-way ANOVA followed by Dunnett’s multiple comparison tests.

Inflammation in the IR myocardium may play a role in the pathogenesis of cardiomyocyte death in IR myocardium [33]. The ED-1-positive monocytes were observed in the IR myocardium 24 hours after reperfusion in vehicle group. Treatment with Pitavastatin-NP, but not with FITC-NP or pitavastatin alone, reduced the expression of MCP-1 and the number of those leukocytes in the IR myocardium (Fig 7). Pitavastatin-NP significantly inhibited activation of NF-B in the ischemic myocardium (Fig 7). Pitavastatin-NP did not affect circulating leukocyte counts after 24 hours after IR (Table 2). We examined TUNEL staining in the infarct-border myocardium as an indicator of apoptosis. TUNEL-positive cells were noted in the infarct-border myocardium at 24 hours of reperfusion in saline-treated control animals. Treatment with Pitavastatin-NP, but not with FITC-NP or pitavastatin alone, reduced the number of TUNEL-positive cardiomyocytes (Fig 7).

### Effects of Pitavastatin-NP on myocardial function and remodeling

**Fig 8:**
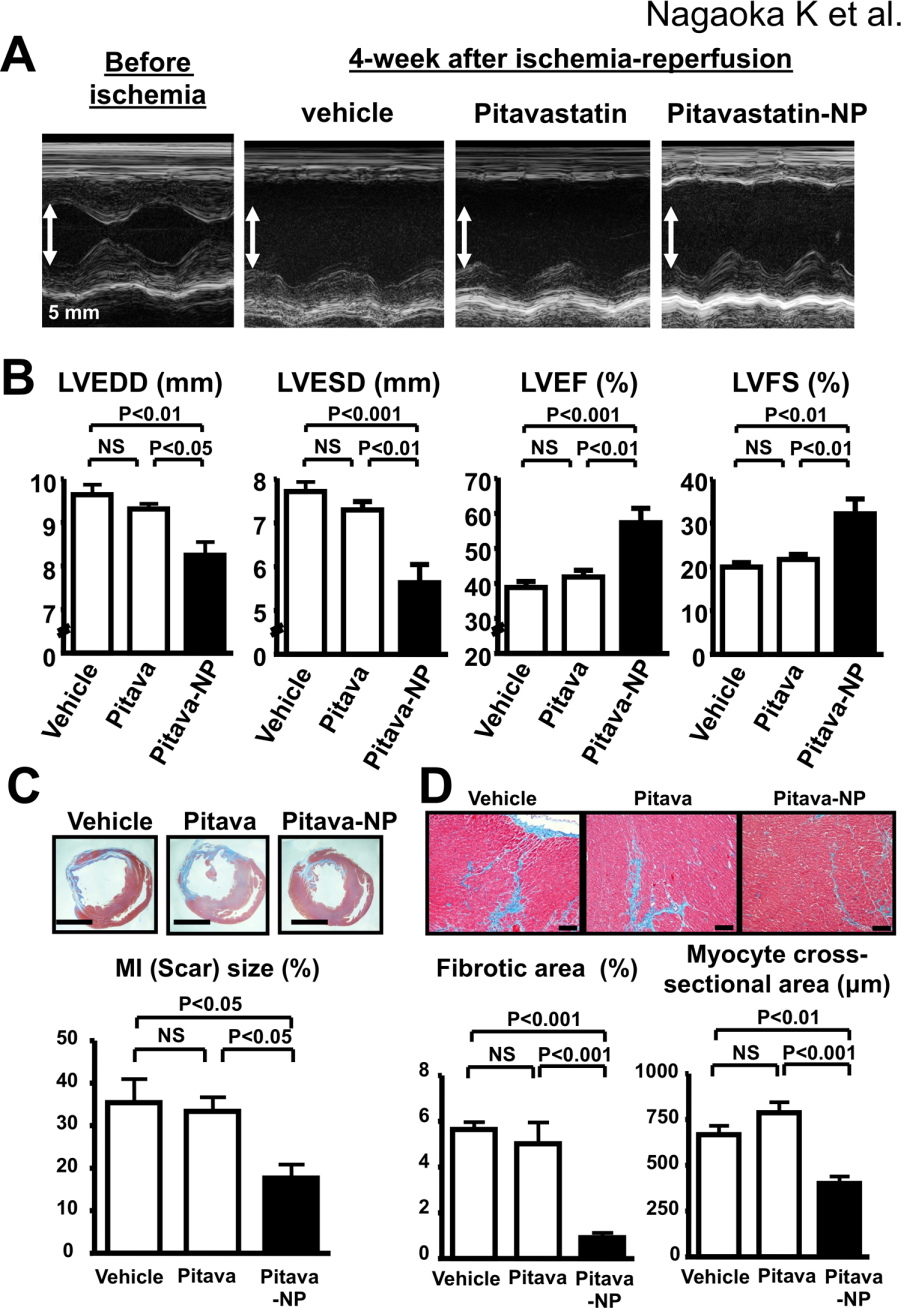
Effects of Pitavastatin-NP on left ventricular remodeling after reperfusion. **(A),** Representative M-mode echocardiograms for animals treated with pitavastatin alone or Pitavastatin-NP 4 weeks after reperfusion. Scale bar: 5 mm. **(B),** Effects of Pitavastatin-NP on LVEDD, LVESD, LVEF and LVFS 4 weeks after reperfusion. N = 8 per group. Data are compared using one-way ANOVA followed by Bonferroni’s multiple comparison tests. **(C)**, Effects of Pitavastatin-NP on the extent of Masson-trichrome-positive scar 4 weeks after reperfusion. N = 5–6 per group. Data are compared using one-way ANOVA followed by Bonferroni’s multiple comparison tests. **(D)**, Effects of Pitavastatin-NP on the extent of fibrosis and cardiomyocyte hypertrophy in the border zone 4 weeks after reperfusion. N = 5–6 per group. Data are compared using one-way ANOVA followed by Bonferroni’s multiple comparison tests.

Echocardiography 4 weeks after IR showed that both of LV end-diastolic diameter (LVEDD) and LV end-systolic diameter (LVESD) were significantly increased in vehicle group. In contrast, intravenous treatment with Pitavastatin-NP at the time of reperfusion, but not pitavastatin alone, reduced the increase in LVEDD and LVESD 2 days, 1 week, 2 weeks, and 4 weeks after IR (Fig 8A and 8B and Table 4). Pitavastatin-NP, but not pitavastatin alone, attenuated the decrease in LV ejection fraction (LVEF) and LV fractional shortening (LVFS) 4 weeks after IR (Fig 8A and 8B and Table 4). Histological analysis showed that Pitavastatin-NP reduced Masson-trichrome-positive scar (Fig 8C), and fibrosis and cardiomyocyte hypertrophy in the border zone (Fig 8D). Treatment with Pitavastatin-NP or pitavastatin alone did not affect systolic blood pressure and heart rate 1 week, 2 weeks, and 4 weeks after IR (Table 5).

**Table 4:** Effects of Pitavastatin-NP on cardiac remodeling measured by echocardiography.

Groups		Weeks after IR				
		Baseline	2 days	1 week	2 weeks	4 weeks
Vehicle	LVEDD (mm)	6.2 ± 0.1	6.9 ± 0.2	8.0 ± 0.2	8.7 ± 0.1	9.6 ± 0.2
	LVESD (mm)	2.6 ± 0.2	4.4 ± 0.1	5.9 ± 0.3	6.8 ± 0.2	7.7 ± 0.2
	LVEF (%)	86.2 ± 1.6	63.1± 2.3	49.5 ± 3.0	42.1 ± 2.6	38.9 ± 1.7
	LVFS (%)	57.1 ± 2.2	35.4 ± 1.9	26.3 ± 1.9	21.8 ± 1.6	20.0 ± 1.0
Pitava	LVEDD (mm)	6.1 ± 0.1	6.7 ± 0.2	7.6 ± 0.2	8.7 ± 0.2	9.3 ± 0.1
	LVESD (mm)	2.7 ± 0.2	4.5 ± 0.2	5.8 ± 0.2	6.6 ± 0.2	7.3 ± 0.2
	LVEF (%)	85.9 ± 1.6	58.6 ± 2.4	46.3 ± 2.3	46.5 ± 2.0	41.9 ± 1.9
	LVFS (%)	56.7 ± 2.0	32.0 ± 1.8	24.1 ± 1.4	24.4 ± 1.2	21.7 ± 1.1
Pitava-NP	LVEDD (mm)	6.2 ± 0.1	6.2 ± 0.1*	7.0 ± 0.2**	7.5 ± 0.1***	8.2 ± 0.3**
	LVESD (mm)	2.7 ± 0.1	3.8 ± 0.2*	4.7 ± 0.2**	5.1 ± 0.3***	5.6 ± 0.4**
	LVEF (%)	86.0 ± 1.6	67.9 ± 2.4	61.0± 3.1*	56.6 ± 4.7*	57.4 ± 4.1***
	LVFS (%)	56.8 ± 2.0	38.9 ± 1.8	34.0 ± 2.2*	31.4 ± 3.4	32.1 ± 3.4**

Data are expressed as the mean ± SEM (n = 8 each).

*P<0.05

**P<0.01, and

***P<0.001 versus vehicle group. Abbreviations: LVEDD, left ventricular end-diastolic diameter; LVESD, left ventricular end-systolic diameter; LVEF, left ventricular ejection fraction; LVFS, left ventricular fractional shortening.

**Table 5:** Effects of pitavastatin-NP on systolic blood pressure and heart rate after IR.

**Systolic BP (mmHg)**				
Groups	Baseline	Weeks after IR		
		1 week	2 weeks	4 weeks
Vehicle	121±3	112±5	116±2	118±3
Pitavastatin	120±3	107±3	116±2	113±3
Pitavastatin-NP	120±3	106±4	111±3	118±3
**HR (bpm)**				
Groups	Baseline	Weeks after IR		
		1 week	2 weeks	4 weeks
Vehicle	394±12	401±14	381±9	405±17
Pitavastatin	405±10	402±10	393±13	423±15
Pitavastatin-NP	379±11	413±19	380±11	405±13

Data are expressed as the mean ± SEM (N = 6–8 each).

## Discussion

The novel findings of the present study are as follows: (1) PLGA nanoparticles are selectively delivered to IR myocardium and inflammatory cells after intravenous injection at the time of reperfusion, (2) PLGA nanoparticle-mediated delivery of pitavastatin (Pitavastatin-NP) protects the hearts from IR injury as seen with reduction in MI size and ameliorated maladaptive LV dysfunction, and (3) Pitavastatin-NP inhibits myocardial inflammation after IR and protects IR hearts independently of mPTP opening.

In the early phase of IR injury, calcium overload and excessive production of reactive oxygen species induces mPTP opening and mitochondrial dysfunction leading to myocardial necrosis [3]. Several studies have shown that the activation of RISK pathway PI3K/Akt is a substantial therapeutic target for cardioprotection from IR injury [25,34,35]. Akt negatively regulates apoptosis signal-regulating kinase 1 (ASK1) in IR cardiomyocytes [36], which may protect IR myocardium from apoptosis [37]. Akt phosphorylates and inactivates GSK3β that contributes to myocardial necrosis in the early phase of reperfusion by mPTP opening. GSK3β also contributes to myocardial apoptosis through multiple mechanisms including the phosphorylation of Bax causing its translocation to the mitochondria [38], the destabilization of pro-survival transcriptional factor β-catenin [39], which cause cardiomyocyte death in the late phase of reperfusion [40]. Sanada et al. [34] have shown that statins administered before the onset of 90-minute ischemia protects cardiomyocytes from IR injury in vivo, via activation of the PI3K and Akt, which is consistent with our present data showing that the PI3K inhibitor, wortmannin, blocked the phosphorylation of Akt, and the therapeutic effect of Pitavastatin-NP (Figs 5A and 6B). In contrast, Kocsis et al. reported that chronic or acute lovastatin treatment reduced phosphorylation of Akt [41]. The difference in mechanism of therapeutic effects of statins might be attributed to class effects of statins, dose of statins, time of drug administration, or duration of ischemia/reperfusion.

In this study, Pitavastatin-NP administered at the time of reperfusion induced phosphorylation of Akt and GSK3β in IR myocardium at later phase (3 hour), but not at early phases (15 and 30 min) after reperfusion (Fig 5A, 5C and 5E). Pitavastatin-NP did not reduce mitochondrial swelling, without any effect on cytochrome c leakage to the cytosol early after IR (a marker of mPTP opening) (Fig 4B and 4D). Importantly, the therapeutic effect of Pitavastatin-NP on MI size was noted in rats pretreated with cyclosporine A, an inhibitor of mPTP opening (Fig 6A). Therefore, these data suggest that the cardioprotective effect of Pitavastatin-NP was independent of mPTP opening, a major mechanism of myocardial IR injury in the early phase of reperfusion.

Previous reports demonstrated that pretreatment with high dose statins 6 hours (mice) [7] or 12 hours (human) [42] before ischemia reduced MI size, while treatment with high dose statin at the time of reperfusion failed to reduce MI size [7,10,11]. In the present study, we demonstrated that intravenous treatment with Pitavastatin-NP containing 1 mg/kg pitavastatin at reperfusion reduced MI size at 24 hours, and ameliorated LV dysfunction and cardiac remodeling, such as fibrosis and hypertrophy in the border area, at 4 weeks after IR. In contrast, intravenous treatment with pitavastatin at doses as high as 10 mg/kg (maximal soluble dose) showed no therapeutic effects on MI size. These data suggest that nanoparticulation is essential for cardioprotection of statins from IR injury in case administered at reperfusion. Fluorescent imaging, flow cytometry, and histopathological analysis showed selective delivery of FITC-NP into cardiomyocytes and monocytes within IR myocardium. A prior report by Takahama et al. [43] has shown that liposome coated with polyethylene glycol (mean diameter 134±21 nm) selectively delivered in cardiomyocytes in the border and infarcted area by electron microscopy in the same rat model, although the precise mechanisms by which liposomal nanoparticles accumulate into IR myocardium was unclear. We found co-localization of FITC-NP and Evans blue signals in the IR myocardium, suggesting that PLGA nanoparticles enhanced drug delivery into IR myocardium at least in part through increased vascular permeability in the IR myocardium in this rat model. Lin et al. has recently shown that reoxygenation of anoxic cardiac myocardium induces massive palmitoylation dependent endocytosis [44], which suggested that cardiomyocytes after reperfusion may uptake nanoparticle by enhanced endocytosis.

Pharmacokinetic analysis (Table 1) suggest that the difference in pitavastatin concentrations of IR myocardium between pitavastatin and pitavastatin-NP groups is not therapeutically relevant. It is unclear whether the observed difference in pitavastatin concentrations in Pitavastatin-NP group of approximately 2- to 3-fold between IR myocardium and non-ischemic myocardium accounts for the superior therapeutic effect of Pitavastatin-NP. Further studies are needed to determine whether the measured tissue concentration truly reflects intracellular concentrations of pitavastatin in IR cardiomyocytes.

It is known that inflammation after IR plays a key role in the extension of myocardial IR injury. Monocytes are protagonists of infarct inflammation and are recruited into IR myocardium mainly via expression of chemokines MCP-1 [33,45–47]. These leukocytes generate reactive oxygen species and stimulate the release of pro-apoptotic factors in the ischemic myocardium, resulting in cardiomyocyte apoptosis after IR [48,49]. Hayasaki et al. reported that blockade of CC chemokine receptor 2 (CCR2), the MCP-1 receptor, attenuates myocardial IR injury in mice [50]. Therefore, MCP-1/CCR-2 pathway may be a feasible therapeutic target to inhibit inflammation after IR and to reduce MI size. In the present study, Pitavastatin-NP effectively inhibited the infiltration of ED-1-positive monocytes into the IR myocardium that was associated with inhibition of NF-B activation that is known to be regulated by GSK3β [51], and expression of MCP-1. These data imply that cardioprotective effect of Pitavastatin-NP depends on the inhibition of the MCP-1/CCR2 pathway via inactivation of NF-B pathway as we have shown in a previous study [14,52], resulting in reduced monocyte-mediated inflammation and cardiomyocyte apoptosis in the late phase of reperfusion in this study.

In summary, nanoparticle-mediated targeting of pitavastatin into IR myocardium induced cardioprotection from IR injury as seen by the reduction in MI size and improvement of LV function via activation of PI3K pathway and inhibition of inflammation and cardiomyocyte death in this model. Because we have produced Pitavastatin-NP according to good manufacturing practice guidelines and completed a phase I clinical trial at Kyushu University Hospital (UMIN 000014940) to investigate the safety of a single intravenous infusion of Pitavastatin-NP in healthy volunteers, this nanoparticle-based technology can be developed as an innovative therapeutic modality for IR injury in hearts and other organs, such as brain, kidney, and liver.
